# Genome-wide association scan and candidate gene analysis for seed coat color in sesame (*Sesamum indicum* L.)

**DOI:** 10.3389/fpls.2025.1541656

**Published:** 2025-01-28

**Authors:** Mohammed Elsafy, Wafa Badawi, Ahmed Ibrahim, Elamin Hafiz Baillo, Prabin Bajgain, Tilal Sayed Abdelhalim, Mahbubjon Rahmatov

**Affiliations:** ^1^ Department of Plant Breeding, Swedish University of Agricultural Sciences (SLU), Alnarp, Sweden; ^2^ Agricultural Research Corporation (ARC), Ministry of Agriculture, Wad Madani, Sudan; ^3^ Department of Agronomy and Plant Genetics, University of Minnesota, SaintPaul, MN, United States; ^4^ Biotechnology and Biosafety Research Center, Agricultural Research Corporation, Shambat, Khartoum North, Sudan

**Keywords:** biosynthesis, encoding proteins, GWAS, pigmentation, Sudan

## Abstract

**Introduction:**

Seed coat color in sesame is a crucial trait for breeding programs as it is closely associated with important characteristics such as oil content, protein levels, and disease resistance, which directly influence seed quality and market value.

**Methods:**

This study investigates the genetic basis of seed coat color in 200 Sudanese sesame genotypes grown for two consecutive years through comprehensive phenotyping, genomic diversity analysis, genome-wide association studies (GWAS), and candidate gene discovery.

**Results and discussion:**

Phenotypic analysis across two growing seasons revealed high heritability and significant correlations among color parameters (L*, a*, and b*), indicating strong genetic control over seed coat color. The genomic analysis identified distinct clusters among sesame accessions, with rapid linkage disequilibrium decay suggesting a high level of recombination. GWAS identified significant SNPs associated with seed coat color traits, revealing key genomic regions on chromosomes 3, 6, 9, 12, and 13. Candidate gene analysis highlighted several genes, including *DOF* zinc finger proteins and *WRKY* transcription factors, which may play essential roles in pigment biosynthesis pathways. These findings provide valuable insights for breeding programs to enhance desirable seed coat color traits in sesame.

## 1 Introduction

Sesame (*Sesamum indicum* L.) is a globally important oilseed crop cultivated in over 70 countries with a production of 6.8 million tons (FAOSTAT, 2022). Sesame is known as the ‘queen of oil crops,’ it is valued for its high oil content (up to 60%) and rich composition of proteins, fatty acids, and antioxidants like sesamin and sesamolin (Dar et al., 2019). Sudan, the largest producer of sesame, is considered a center of origin for this crop, making it a crucial location for genetic diversity studies and breeding efforts (Idris et al., 2023).

Among the various traits of interest in sesame breeding, seed coat color has emerged as a critical characteristic influencing consumer preferences and potential health benefits. However, sesame seeds exhibit a wide range of colors, including white, yellow, brown, and black, primarily determined by the accumulation of pigments such as chlorophyll, carotenoids, and various phenolic compounds in the seed coat (Wang et al., 2016). Recent studies have highlighted the complex relationship between seed coat color and agronomically important traits in sesame. For instance, black sesame seeds contain significantly higher levels of phenolic compounds and exhibit greater antioxidant activity than white seeds (Mi et al., 2022). Darker seeds also have higher concentrations of lignans, particularly sesamin and sesamolin, which are known for their health-promoting properties (Abbas et al., 2022). Regarding disease resistance, pigmented seed coats are associated with enhanced protection against pathogens, such as increased resistance to Fusarium wilt (Dutta et al., 2022). Additionally, seed coat color influences oil content and fatty acid composition, as white-seed varieties generally have higher oil content, while black-seed varieties often exhibit a more favorable fatty acid profile (Uzun et al., 2008; Wei et al., 2015).

The genetic basis of key traits in sesame has become easier to study due to its relatively small diploid genome (2*n* = 26), estimated at 357 Mb (Wang et al., 2016). Sequencing of the sesame genome (Wang et al., 2014) and its improved assembly and annotation (Wang et al., 2022) have greatly advanced genetic research. Recent advances in genomic technologies, particularly genome-wide association studies (GWAS), have identified genetic loci associated with traits such as oil content, fatty acid composition, and disease resistance (Wei et al., 2015; Zhao et al., 2022; Zhou et al., 2022). A previous GWAS on seed coat color in sesame identified 13 significant single nucleotide polymorphisms (SNPs) associated with this trait, including a major locus on LG4 harboring the *PPO* gene involved in melanin biosynthesis (Wei et al., 2015). Cui et al. (2021) identified 197 significant SNPs associated with seed coat color, including 30 detected across six environments and 92 candidate genes located near four of these SNPs. However, due to the complexity of seed coat color and its links to various biochemical and agronomic traits, further research is needed to understand its genetic architecture.

Understanding the genetic control of seed coat color is essential for developing cultivars with desired characteristics to meet diverse market demands. White-seed sesame is widely preferred in many markets for its perceived quality and value (Uzun et al., 2008), while black sesame is gaining popularity due to its higher antioxidant content and potential health benefits (Abbas et al., 2022). The CIELab color space, measuring lightness (L*), redness-greenness (a*), and yellowness-blueness (b*), provides a standardized method for quantifying seed coat color (Pathare et al., 2013). It has been widely recognized for its effectiveness in seed color characterization, developing a device-independent method that provides superior perception accuracy compared to Red, Green, and Blue (RGB) models (Ivanova et al., 2022). However, RGB frequently lacks the precision needed for subtle variations, which is essential in agricultural applications (Dong et al., 2018). It has been documented that CIELab is reliable in evaluating seed quality and vigor in various crop species (Armoniené et al., 2018). In addition, CIELab has been validated in other contexts, including food science and medical imaging (Dong et al., 2018).

In this study, we build upon previous GWAS efforts by leveraging a more comprehensive and genetically diverse sesame panel, combined with high-density SNP markers, to elucidate the genetic architecture underlying seed coat color. Advanced phenotyping approaches were employed to measure CIELab color parameters (L*, a*, and b*), providing a detailed assessment of seed coat color variation and enabling comprehensive analysis of its genetic contributors. The findings from this study will contribute to our understanding of pigment biosynthesis in sesame and provide valuable information for breeding programs aimed at tailoring seed coat color to meet market and nutritional demands. This study will enhance our understanding of sesame genetic diversity in Sudan, a major center of its origin, and may identify new alleles associated with seed coat color and other important traits.

## 2 Materials and methods

### 2.1 Experiment setup

The field trial was conducted over two consecutive growing seasons (2021 and 2022) at the Matuq Research Station in Gaziera State, Sudan (14°11’10”N, 32°34’48”E). A total of 200 genetically diverse sesame accessions, along with 3 control checks, were evaluated using an augmented block design. The experimental layout consisted of 8 blocks, comprising 28 plots. Within each block, 25 distinct accessions were randomly assigned, and the 3 check varieties were replicated across all blocks to provide a measure of inter-block variability.

Standard agronomic practices tailored to local conditions were carefully followed throughout the growing season. These practices included appropriate land preparation, timely sowing, optimal irrigation scheduling, and recommended fertilizer application rates. Pest and disease management were carried out as needed to ensure healthy plant growth and development. Upon reaching physiological maturity, the sesame plants were harvested manually, and seeds were carefully extracted, cleaned, and dried to a uniform moisture content. Subsequently, the seed samples were stored under controlled environmental conditions to maintain seed quality and viability until laboratory analysis could be performed.

### 2.2 Seed coat color measurement and data analysis

Seed coat color parameters were quantified using (Chroma Meter CR 400, manufactured by Minolta, Japan). This device measures color in the CIELab color space, where L* represents lightness (0 = black, 100 = white), a* indicates the red-green spectrum (-60 = green, +60 = red), and b* denotes the blue-yellow spectrum (-60 = blue, +60 = yellow). Before measurements, the colorimeter was calibrated using a standard white reflector plate (Y = 93.7, x = 0.3160, y = 0.3323) to ensure accuracy. For each sample, 50.0 g of sesame seeds were carefully placed into a clean, dry Petri dish attached to the colorimeter, ensuring a uniform layer with complete coverage of the measurement area. Three replicate measurements were taken for each sample, rotating the Petri dish 120° between readings to account for any potential heterogeneity in seed color distribution (Elsafy et al., 2024).

### 2.3 Statistical analyses

The relationships and distributions of color traits across two years (2021 and 2022) were examined using correlation analysis and scatter plots in the R ‘ *GGally*’ package (Schloerke et al., 2021). The Principal Component Analysis (PCA) biplot for the seed coat color attributes was created using ‘*factoextra*’ R package (Kassambara and Mundt, 2016).

The broad-sense heritability (H) for sesame seed coat color was calculated using:


$$H=\sigma_{G}^{2}/(\sigma_{G}^{2}+\frac{\sigma_{GL}^{2}}{L}+\frac{\sigma_{E}^{2}}{L})$$

Where $\sigma_{G}^{2}$ is represents the genetic variance, $\sigma_{GL}^{2}$ is denotes the genotype by year interaction variance, $\sigma_{E}^{2}$ is the residual from environmental variance, and *L* is the number of years.

### 2.4 Genetic material preparation and sequencing

From each line, a circular section of young leaf tissue, approximately 5 mm in diameter, was harvested from each plant and placed into a 96-well plate designed for tissue collection. Genomic DNA was extracted using the Qiagen BioSprint 96 system alongside the Qiagen BioSprint DNA Plant kit. DNA was normalized to ng/μL concentration, and sequencing libraries were prepared using a genotyping-by-sequencing (GBS) protocol (Poland et al., 2012). Specifically, the restriction enzymes *PstI* and *MspI* were used to induce cuts at multiple sites in the genome, and the resulting pool was ligated with unique barcode adapters, multiplexed with 96 samples per lane, sequenced on a NovaSeq 6000 (Illumina, San Diego, CA, USA). Sequencing of the DNA libraries was done at the University of Minnesota Genomics Center (St. Paul, MN, USA).

Generated sequencing data was filtered for a minimum quality (Q) score of 30 and demultiplexed using ‘*sabre*’ (https://github.com/najoshi/sabre) to sort separate reads corresponding to each sample. The reads were then aligned to the *Sesamum indicum* updated genome assembly and annotations (Wang et al., 2022) with the Burrow-Wheelers Alignment (BWA) tool version 0.7.4 (Li and Durbin, 2009). Genome-wide SNPs were identified using Samtools and bcftools (Li, 2011). The SNP markers were filtered to retain those with a minimum minor allele frequency (MAF) of 3% and a missing allelic proportion of 20% or less. This resulted in 3,636 SNPs distributed among the 13 chromosomes and 17 high-confidence scaffolds.

### 2.5 Genetic diversity, population structure, and marker density analysis

Genetic relationships among accessions were assessed using TASSEL v5.2.60 (Bradbury et al., 2007). The genetic similarity matrix was computed using the identity-by-state (IBS) algorithm with 10,000 bootstraps. The resulting matrix was visualized as a heatmap using the ‘*heatmap*’ package in R (Kolde and Kolde, 2015). The Bayesian clustering approach was implemented to elucidate population structure using ADMIXTURE v1.3.0 (Alexander et al., 2009). The optimal number of ancestral populations (K) was determined by running the analysis for K values ranging from 1 to 10, with 10 independent runs for each K. The best K was selected based on the lowest cross-validation error. Results were visualized using the ‘*pophelper*’ R package (Francis, 2017). To evaluate LD decay, pairwise linkage disequilibrium between markers was calculated using Tassel 5, utilizing a sliding window method with 50 markers. The LD values, represented as *r* ^2^, were graphed against physical distances derived from the Sesame genome V.3.0 reference. A locally weighted scatterplot smoothing (LOWESS) curve was used to visualize the LD decay pattern, and the LD decay distance was estimated following the method described by Hill and Weir (1988), and the SNP linkage disequilibrium (LD) heatmap physical length and the number of SNPs within 1Mb were estimated using the SRPlot interface (Tang et al., 2023).

### 2.6 Seed coat color traits association and candidate gene search

GWAS was conducted using GAPIT 3 in R 4.3.2, employing the Fixed and random model Circulating Probability Unification (FarmCPU) method (Liu et al., 2016; Wang and Zhang, 2021). Significant markers were identified based on the Bonferroni-corrected threshold (α = 0.01). For individual SNPs, this corresponded to a *p*-value of roughly determined using a *cutoff* calculated as the total number of markers (3636) divided by 1000. This yielded a threshold corresponding to a logarithm of the odds (LOD) score of approximately 3, which is presented as Manhattan and QQ plots.

Searching for candidate genes was conducted by examining the regions surrounding significant SNP markers to identify genes potentially influencing seed coat color. We analyzed protein-coding sequences within 409,780 bp of significant loci based on the average linkage disequilibrium in sesame (204,890 bp). Using a refined sesame genome assembly (Wang et al., 2022), we conducted a protein BLAST search on the NCBI clustered nr database (Coordinators, 2015). We focused on *Sesamum indicum* sequences with >80% identity and E-values ≤1E-10, retaining the top three alignments for each sequence. These were then filtered to identify candidate genes that regulate seed coat color.

## 3 Results

### 3.1 Seed coat color phenotyping

**Figure 1:**
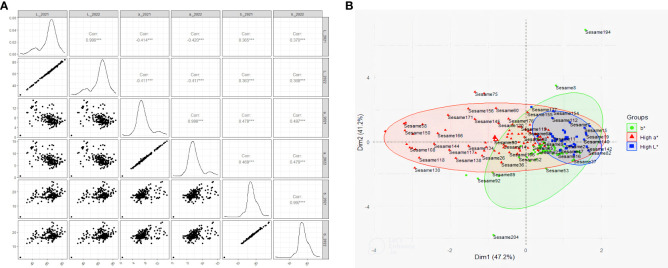
**(A)** Correlation of seed coat color traits of the sesame accessions across two consecutive seasons, 2021 and 2022. **(B)** Principle Component Analysis (PCA) of seed coat color traits of the sesame accessions.

Analysis of sesame seed colorimetric parameters (L*, a*, and b*) across two growing seasons (2021 and 2022) revealed exceptionally high consistency between years (*r* = 0.997, p < 0.001), indicating solid genetic control over seed coat color (**Figure 1A**). The lightness (L*) values demonstrated the most comprehensive range, suggesting significant variability in seed coat brightness across accessions. Importantly, we observed moderate negative correlations between L* and a* values (*r* = -0.42, p < 0.001) and moderate positive correlations between L* and b* values (r = 0.37, p < 0.001), which indicates that lighter seeds tend to be less red but more yellow. The a* and b* values showed moderate positive correlations (*r* = 0.47, p < 0.001), indicating that reddish seeds also tend to be more yellow. On the other hand, the PCA showed significant insights into seed coat color variation, where the first two principal components account for a substantial 88.4% of the total variance of Dim1: 47.2%, Dim2: 41.2% (**Figure 1B**). Three distinct groups emerge a high b* group characterized by greater yellowness, a high a* group showing increased redness, and a high L* group representing lighter seeds. Interestingly, considerable overlap exists between the high L* and high b* groups, showing a positive correlation between seed lightness and yellowness. The broad sense heritability estimates for the seed coat color traits were remarkably high, with H values of 0.9991, 0.9975, and 0.9974 for L*, a*, and b*, respectively.

### 3.2 Genetic diversity and structure analysis

**Figure 2:**
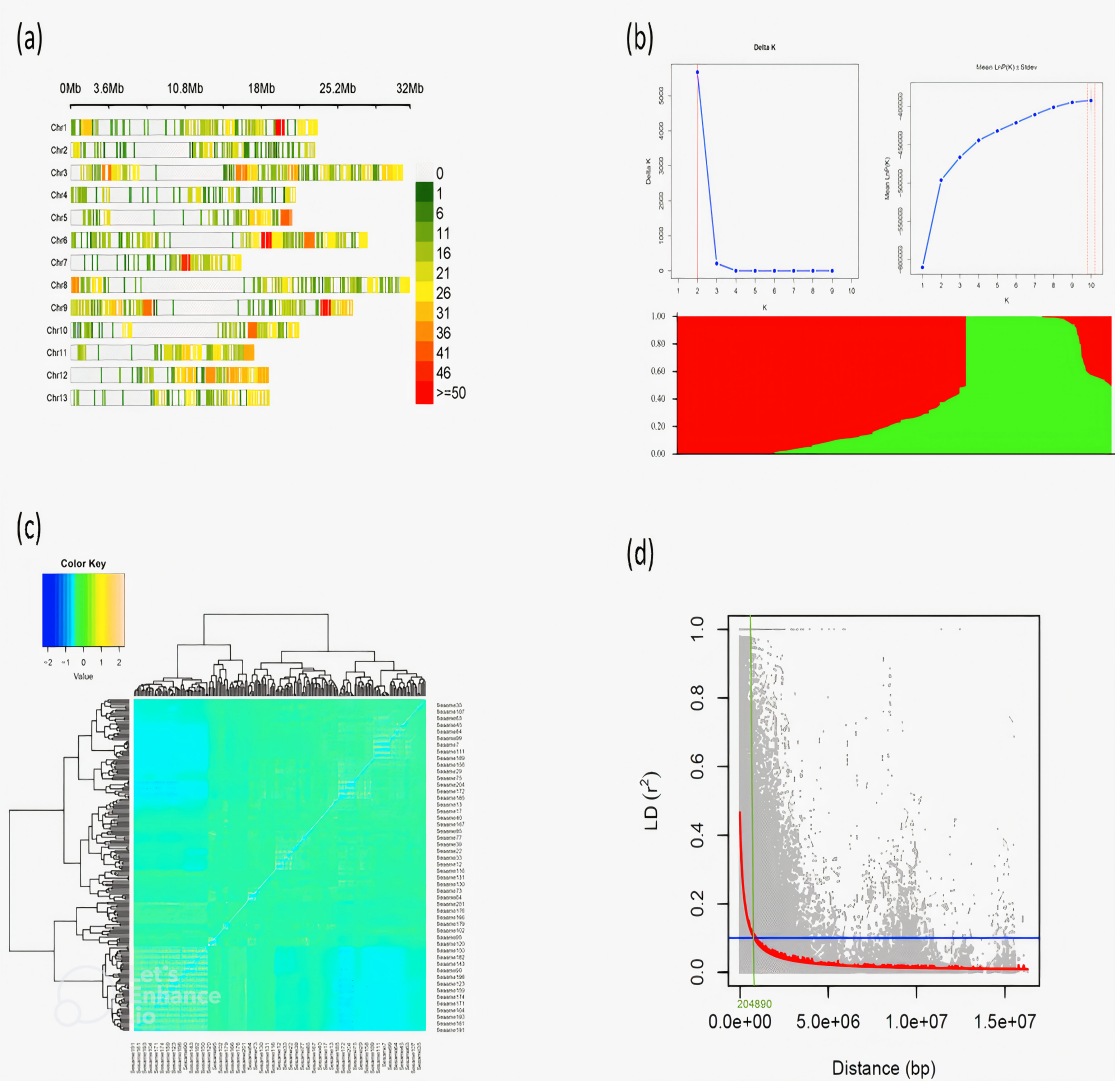
**(A)** Marker density across the 13 chromosomes of the sesame accessions. **(B)** Population structure analysis estimated by Delta K and LnP(K) values and heatmap shows the structure of subpopulations in sesame accessions. **(C)** Kinship heatmap showing the population relationships among the sesame accessions based on additive relationships. **(D)** linkage disequilibrium (LD) decay across genomic distance in a population of sesame accessions.

The genomic landscape of the sesame accessions is characterized by a heterogeneous distribution of genetic variants across the 13 chromosomes (**Figure 2A**). Chromosomes 5, 7, and 10 exhibit regions of high variant density, as indicated by the red and orange bands. Population structure analysis (**Figure 2B**) reveals a significant division of sesame accessions into two distinct clusters, as determined by the ΔK method, with the optimal K = 2 indicating clear genetic differentiation. Admixture analysis further supports this, showing distinct proportions of genotype patterns between the two subpopulations. The kinship heatmap (**Figure 2C**) illustrates the genetic relatedness among the sesame accessions, with distinct blocks indicating varying degrees of relatedness, highlighting the presence of genetically similar groups within the sesame population. Linkage disequilibrium (LD) decay analysis (**Figure 2D**) demonstrates a rapid decline in LD with increasing physical distance, with an *r* ^2^ value of 0.1 at a physical distance of approximately 0.204 Mb.

### 3.3 Seed coat color traits association

**Figure 3:**
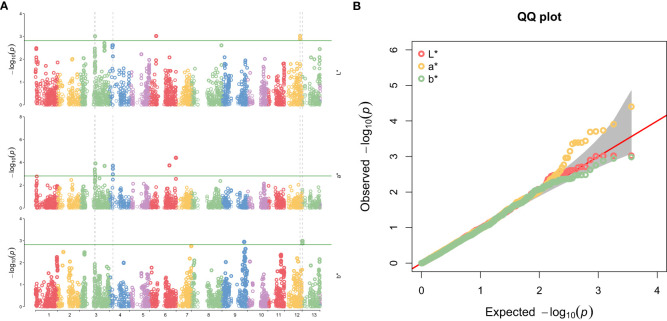
**(A)** Manhattan Plot for SNPs associations with seed coat color traits (L*, a*, and b*) in sesame accessions. **(B)** QQ plot showing deviation from the null hypothesis of associated seed coat color traits.

GWAS identified several SNPs associated with the seed coat color traits L*, a*, and b* (**Table 1**) and Manhattan plot (**Figure 3A**). For the L* trait, significant associations were detected on chromosomes 3, 6, and 12. The most relevant markers were located at positions 16,523,829 bp and 16,523,899 bp on chromosome 12 (allele G/A), with a p-value of 0.0009, explaining 6.51% of the phenotypic variance. For a* trait, significant SNPs were predominantly located on chromosome 3, including the marker at position 15960455 bp (allele C/A) with a p-value of 0.0004, explaining 7.17% of the phenotypic variance. Additionally, a highly significant marker was identified on chromosome 6 at position 27694080 bp (allele T/G) with a p-value of less than 0.0001, accounting for 9.20% of the phenotypic variance. For the b* trait, significant associations were identified on chromosomes 9 and 13. The SNPs at positions 345,249 bp (allele G/A) and 345,322 bp (allele T/G) on chromosome 13 showed strong associations with p-values of 0.0013 and 0.0010, respectively, explaining over 6% of the phenotypic variance. This genetic association for seed coat color traits was confirmed by the QQ plot (**Figure 3B**), which considered population structure and quality control factors.

**Table 1:** Genome-wide detection of genetic markers associated with seed coat color traits in 200 sesame accessions.

Trait	SNP marker	Chr	Pos (bps)	Alleles	p-value	LOD	MAF	R² (%)	Allelic effect
L*	*Chr12_16523829*	12	16523829	G/A	0.0009	3.0225	0.34	6.51	-3.80
L*	*Chr12_16523891*	12	16523891	C/T	0.0013	2.9002	0.33	6.26	3.73
L*	*Chr12_16523899*	12	16523899	G/A	0.0009	3.0225	0.34	6.51	-3.80
L*	*Chr3_16244425*	3	16244425	A/G	0.0010	3.0098	0.13	6.48	5.11
L*	*Chr6_6974622*	6	6974622	G/A	0.0010	3.0175	0.10	6.50	5.58
a*	*Chr3_15951803*	3	15951803	T/C	0.0007	3.1481	0.06	6.76	1.23
a*	*Chr3_15960455*	3	15960455	C/A	0.0004	3.3560	0.05	7.17	1.36
a*	*Chr3_15984070*	3	15984070	A/G	0.0004	3.3909	0.06	7.24	-1.27
a*	*Chr3_15984721*	3	15984721	A/G	0.0004	3.3909	0.06	7.24	-1.27
a*	*Chr3_15984975*	3	15984975	A/G	0.0004	3.3909	0.06	7.24	-1.27
a*	*Chr3_16249093*	3	16249093	G/C	0.0011	2.9629	0.06	6.39	1.13
a*	*Chr3_16593829*	3	16593829	C/A	0.0001	3.9078	0.13	8.26	-0.98
a*	*Chr3_26242291*	3	26242291	C/T	0.0002	3.6894	0.25	7.83	-0.74
a*	*Chr4_3654235*	4	3654235	A/C	0.0004	3.4379	0.18	7.34	1.03
a*	*Chr4_3654271*	4	3654271	G/A	0.0011	2.9605	0.18	6.38	-0.96
a*	*Chr4_3654307*	4	3654307	T/A	0.0002	3.7045	0.17	7.86	-1.10
a*	*Chr6_20862610*	6	20862610	A/G	0.0002	3.7350	0.19	7.92	0.76
a*	*Chr6_27694080*	6	27694080	T/G	0.0040	4.4023	0.04	9.20	-1.66
b*	*Chr13_345249*	13	345249	G/A	0.0013	2.8810	0.02	6.22	-4.59
b*	*Chr13_345322*	13	345322	T/G	0.0010	2.9834	0.06	6.43	-2.60
b*	*Chr9_23287055*	9	23287055	G/A	0.0011	2.9439	0.35	6.35	2.24

This table shows the details of single nucleotide polymorphism (SNP) markers significantly associated with key traits, including L*, a*, and b*, in sesame accessions. The information provided includes the SNP marker ID, (Chr) chromosome and (Position/bps) physical position of the marker, the alleles, (MAF)minor allele frequency, (LOD) logarithm of odds score, (R^2^%) proportion of phenotypic variance explained, and the estimated effect size of the associated allele.

### 3.4 Searching for candidate genes

The candidate gene searching analysis identified several key genes associated with seed coat color space traits in this study (**Table 2**). *APMJ01001391*, encoding the *DOF3.1* zinc finger protein (80.7% identity), and *APMJ01001731*, encoding STY8-like isoform X2 (100% identity), were annotated on chromosome 3, both highly associated with trait a*. On chromosome 6, two genes were identified: *APMJ01003628*, encoding histidine-containing phosphotransfer protein 4-like isoform X2 (100% identity), associated with trait L*, and *KAK4407764*, encoding serine/threonine-protein kinase *STY8* (98.2% identity), associated with trait a*. As a result, all genes identified were found in *Sesamum indicium* except *KAK4407764*, which was found in *Sesamum angolense*. Chromosome 6 also contains *APMJ01003151*, encoding *WRKY* transcription factor 23 (96% identity). Further, *APMJ01007050*, encoding salicylic acid-binding protein 2 (98.5% identity), on chromosome 9 was associated with trait b*, while *APMJ01006505*, encoding Squamosa promoter-binding protein 1 (99.2% identity), on chromosome 12, was linked to trait L*.

**Table 2:** Identified genes associated with L*, a*, and b*in sesame accessions.

NCBI candidate genes	Trait	SNP	Species	Annotation	E-value	% identity
*APMJ01001391*	a*	*Chr3_15984975*	*Sesamum indicum*	dof zinc finger protein DOF3.1-like	3e-123	80.7%
*APMJ01001731*	a*	*Chr3_26242291*	*Sesamum indicum*	serine/threonine-protein kinase STY8-like isoform X2	0.0	100%
*APMJ01003628*	L*	*Chr6_6974622*	*Sesamum indicum*	histidine-containing phosphotransfer protein 4-like isoform X2	2e-99	100%
*KAK4407764*	a*	*Chr6_20862610*	*Sesamum angolense*	Serine/threonine-protein kinase STY8	0.0	98-2%
*APMJ01003151*	a*	*Chr6_27694080*	*Sesamum indicum*	probable WRKY transcription factor 23	2e71	96%
*APMJ01007050*	b*	*Chr9_23287055*	*Sesamum indicum*	salicylic acid-binding protein 2	2e-89	98.5%
*APMJ01006505*	L*	*Chr12_16523829*	*Sesamum indicum*	Squamosa promoter-binding protein 1	1e-88	99.2%

## 4 Discussion

This study underlines the significant genetic basis of sesame seed coat color, as demonstrated by high heritability estimates for traits L*, a*, and b*. These findings confirm that seed coat color is stable across environments, with minimal influence from external factors, making it predominantly controlled by genetic factors. Such stability is essential for breeding programs, ensuring consistency in expressing traits across various cultivation conditions. Significant SNPs associated with the L* trait on chromosomes 3, 6, and 12, and with the b* trait on chromosomes 9 and 13, mark genomic regions of interest for further research. These loci, which explain a substantial proportion of phenotypic variance, provide a foundation for breeding strategies to tailor seed coat colors to specific market demands.

### 4.1 Seed coat color phenotyping

Our study demonstrated high year-to-year consistency in sesame seed coat color (r = 0.997, p < 0.001), indicating that the trait is stable, heritable, and minimally influenced by the environment. This finding aligns with previous research identifying key quantitative trait loci (QTLs) associated with seed coat color in sesame. Du et al. (2019) developed a high-density genetic map and found that seed coat color is primarily influenced by a few major genes and several QTLs, which significantly contribute to its heritability. Because sesame domestication has led to lighter seed colors, which are largely determined by genetic loci (Wei et al., 2016). Results demonstrate that the first two principal components account for 88.4% of the total variance in sesame seed coat color among Sudanese genotypes, indicating a high level of genetic variation within these traits. Based on the substantial variance explained by these components, a limited number of genetic factors are responsible for seed coat color, and distinct phenotypic groups can be formed, explaining genetic differentiation between genotypes. Our study revealed exceptionally high heritability (H) for L*, a*, and b* values, indicating that genetic factors rather than environmental influences predominantly control seed coat color traits. This finding aligns with previous studies, such as Wang et al. (2016), which reported on the fine mapping of plant height and seed coat color quantitative trait loci (QTLs) using a new high-density genetic map, and Du et al. (2019), who constructed a high-density genetic map using specific length amplified fragment (SLAF) sequencing and conducted QTL mapping of seed-related traits in sesame. Building on this, Sabag et al. (2021) emphasized that straightforward selection strategies can significantly enhance the genetic architecture of sesame seed coat color. Furthermore, since these traits are primarily governed by additive genetic variance, Cui et al. (2021) suggested that selection for these traits in breeding programs could be highly effective.

### 4.2 Genomic diversity and structure analysis

This study highlighted the heterogeneous distribution of genetic variants across the sesame chromosomes, particularly the high variant density on chromosomes 5, 7, and 10, provided a valuable framework for understanding the genetic basis of seed coat color and other agronomic traits using improved assembly and annotation of the sesame genome. The findings from various studies underscore the potential for utilizing this genetic information in breeding programs to enhance sesame quality and yield, and future research should continue to explore the functional implications of these genetic variants and their interactions with environmental factors to optimize sesame cultivation (Wu et al., 2014; Zhao et al., 2022; Zhou et al., 2022).

Our study revealed significant separation into two distinct clusters among sesame accessions, as determined by the ΔK method, which provides valuable insights into the genetic diversity and evolutionary history of sesame where hybridization and gene flow between populations can occur. Understanding the population structure and genetic differentiation among sesame accessions is critical to improving specific traits through breeding programs, such as seed coat color (Pandey et al., 2013). Strategically selecting parental lines representing diverse genetic backgrounds by identifying distinct genetic clusters can enhance hybrid vigor and trait improvement. Further, population structure can be used to conserve genetic resources, making it easier for future breeding efforts to preserve various genetic materials (Dossa et al., 2016). The kinship heatmap result in this study showed a visual representation of the genetic relationships among the sesame accessions, where distinct blocks indicate varying degrees of genetic similarity. Such a heatmap is instrumental in identifying clusters of closely related accessions, which can indicate shared ancestry or common breeding practices (Eynard et al., 2016).

This study found that LD decay was rapid with increasing physical distance, which provides insight into sesame’s genetic architecture. LD describes the non-random association of alleles at different loci, and its decay over distance can provide insight into recombination rates and the dynamics of historical populations. The observed *r*² value of 0.1 at a physical distance of approximately 0.204 Mb in this study indicates a relatively short-lived linkage disequilibrium (LD) in sesame, suggesting a high level of recombination within the genome. Comparatively, previous studies using the first version of the sesame genome (Wang et al., 2014) have reported varying patterns of LD in different sesame populations compared to our study that utilized the updated version of the sesame genome (Wang et al., 2022). For instance, Wang et al. (2014) found that LD in sesame decayed to an *r*² of 0.15 over a distance of around 150 kb, indicating the same trend of rapid decay but over a longer distance. According to Du et al. (2019), LD in a diverse set of sesame accessions exhibits a significant decline, with *r*² values dropping to around 0.1 at distances exceeding 100 kb. This finding is consistent with the short-term nature of LD in this study.

According to these comparisons, LD decay in sesame populations can vary. However, the trend still indicates a high level of recombination, and this will benefit breeding programs because it facilitates the introduction of genetic diversity and the selection of desirable traits. The LD decay results have important implications for breeding and genetic studies in sesame. For instance, Francia et al. (2005) and Dubcovsky (2004) suggested that rapid LD decay marker-assisted selection (MAS) could be effectively employed in breeding programs, as the genetic markers associated with desirable traits are likely to be closely linked to the target genes. However, The LDs observed in this study were relatively short, supporting the potential for efficient marker-assisted selection strategies in sesame breeding.

### 4.3 SNPs-trait association analysis

Our study identified significant associations for the L* trait on chromosomes 3, 6, and 12, with the strongest markers on chromosome 12 at positions 16523829 bp and 16523899 bp. These findings partially align with previous reports, such as Wang et al. (2020), where transcriptome analysis identified genes associated with flavonoid biosynthesis pathways involved in light pigmentation​. Similarly, Li et al. (2021) identified QTL hotspots for L* on chromosome 12 using an F_2_ population derived from Chinese accessions​. Another study by Cui et al. (2021) focused on brown seed coat traits primarily associated significant SNPs with chromosome 6​, while this study identifies both chromosome 6 and 12 loci for the L* trait. This difference might be attributable to the genetic diversity of the Sudanese sesame germplasm and the different reference genome used in this study (Wang et al., 2022). Moreover, the Sudanese sesame accessions, known for their adaptation to arid climates, may harbor unique alleles shaped by local selective pressures​.

The GWAS results in this study identified significant SNPs for a* trait on chromosome 3, with a highly significant marker on chromosome 6 (position 27694080 bp) explaining 9.20% of the phenotypic variance. This finding aligns with the work of Wang et al. (2023), where major-effect QTLs for red pigmentation traits were also mapped to a 1.19 Mb interval on chromosome 6​ (qBSCchr6). Additionally, genes associated with anthocyanin biosynthesis pathways, such as MYB and bHLH transcription factors, were identified in several studies as contributors to red pigmentation​​. While chromosome 3 emerged as a key region in this study, it is less frequently highlighted in Wang et al. (2023) and Dutta et al. (2022) using Chinese or Indian germplasm​​. Moreover, the SNPs on chromosome 6 in this study account for higher phenotypic variance than those reported in Cui et al. (2021) and Li et al. (2021), and this could be attributed to the distinct geographic origin of the Sudanese accessions likely plays a role. Different selective pressures, such as high UV exposure, may affect anthocyanin pigmentation in Sudanese sesame, leading to a stronger association on chromosome 3. Additionally, differences in linkage disequilibrium (LD) patterns between populations may result in unique SNP-trait associations in this study compared to those in Asian germplasm.

For the b* trait, our study revealed significant associations on chromosomes 9 and 13 were identified, with markers on chromosome 13 (positions 345249 bp and 345322 bp) showing strong associations. These findings are consistent with prior studies, such as Cui et al. (2021), which identified regions on chromosome 9 linked to seed coat pigmentation​. Transcriptomic studies have further implicated pathways like carotenoid biosynthesis in yellow pigmentation traits​. However, this study identified chromosome 13 markers as key contributors to b*, which is unique compared to previous studies (Cui et al., 2021; Du et al., 2019). Most studies highlight chromosomes 6 and 9 as major contributors to yellow pigmentation in non-Sudanese sesame. However​​, The prominence of chromosome 13 in our study may reflect unique genetic adaptations of Sudanese accessions. Environmental stressors such as drought and heat in Sudan may have shaped seed coat characteristics, favoring alleles in less prominent loci. The local cultural preferences for seed coat colors could also indirectly influence breeding practices, leading to unique allele frequencies​​.

### 4.4 Searching for candidate genes

This study identified candidate genes likely to play important roles in modulating pigment-related traits via their involvement in regulatory and signaling pathways. Their functional characterization could uncover the molecular basis of pigmentation in *Sesamum indicum* and related species. The *DOF Zinc Finger* Protein encoded by *APMJ01001391* has been implicated in regulating light-responsive genes. Iorizzo et al. (2019) stated that the effects of light on flavonoid biosynthesis are crucial as light triggers the production of anthocyanins. As a result of its association with trait a*, this gene might be involved in modulating pigmentation by activating flavonoid pathway genes.

Phosphorylation-dependent signaling pathways require Serine/Threonine Kinases identified in *APMJ01001731* and *KAK4407764*, and Kinases such as *STY8* control carotenoid metabolism and plastid biogenesis (Mazur et al., 2021). This strong correlation indicates that these genes may regulate carotenoid synthesis or other pigment-related processes. Regarding *WRKY*, transcription factors, such as *WRKY23* encoded by *APMJ01003151*, play a critical role in secondary metabolism regulation under stress conditions (Meraj et al., 2020). As anthocyanin serves as a protective pigment, *WRKY23* may modulate anthocyanin biosynthesis under environmental stress. According to Chen et al. (2019), *WRKY* transcription factors are critical for stress-induced pigment accumulation in different species.

Histidine-containing phosphotransfer proteins (*HPTs*) are essential in the cytokinin signaling pathway. Cytokinin can affect pigment biosynthesis by affecting plasid development and secondary metabolite pathways (Cortleven and Schmülling, 2015). It also aligns with findings from other studies, where cytokinin signaling indirectly affects pigment accumulation through developmental cues (Wu et al., 2021). Furthermore, *SABP2*, encoded by *APMJ01007050*, is implicated in signal transduction in salicylic acid pathways, influencing flavonoid biosynthesis during biotic and abiotic stresses (Shaukat et al., 2022). This trait may mediate stress-mediated pigment accumulation due to its association with trait b*. One of the most notable discoveries is the identification of Squamosa Promoter-Binding Protein 1 (*SBP1*) encoded by *APMJ01006505*, which is linked to trait L*. According to Sánchez-Retuerta et al. (2018), *SBP* proteins regulate gene expression via light-mediated pigment pathways, and because of their high identity and significant annotation, *SBP1* may directly regulate genes involved in carotenoid biosynthesis.

### 4.5 Implications on breeding

The findings from this study have significant implications for plant breeding, particularly for improving desirable agronomic characteristics. The identification of high heritability and strong genetic correlations among color parameters (L*, a*, and b*) suggests that seed coat color can be effectively selected for breeding programs, facilitating the improvement of oil content and disease resistance traits that are closely associated with color. GWAS results identified significant SNPs linked to seed coat color traits, which can be used for marker-assisted selection to accelerate breeding. Moreover, discovering genes involved in pigment biosynthesis pathways, such as DOF zinc finger proteins and WRKY transcription factors, creates new opportunities for genetic manipulation and targeted breeding methods to optimize seed quality. The findings enhance our understanding of the genetic architecture of seed coat color and provide a strong framework for developing sesame varieties with improved market value and agronomic performance.

## 5 Conclusions

The results of this study underscore the importance of genetic factors in determining seed coat color in sesame, with high heritability estimates confirming the stability of these traits across environments. Identifying significant SNPs associated with color traits offers potential markers for marker-assisted selection in breeding programs. Furthermore, the discovery of candidate genes involved in pigment biosynthesis pathways provides a foundation for future functional studies to elucidate the molecular mechanisms underlying seed coat color variation. This study contributes to understanding genetic diversity in sesame and highlights the potential for targeted breeding strategies to improve seed quality and marketability based on color traits. Future studies should focus on the functional characterization of the identified candidate genes and their interactions with the environment to optimize sesame cultivation and enhance its agronomic value.

## Data Availability

The datasets presented in this study can be found in online repositories. The names of the repository/repositories and accession number(s) can be found below: https://www.ncbi.nlm.nih.gov/, PRJNA1184775.
